# Surgical alteration of uterine space influences embryonic loss and fetal growth in the contemporary pig

**DOI:** 10.1186/s12917-025-04820-x

**Published:** 2025-05-20

**Authors:** Dayeon Jeon, Alyssa A. Smith, Sarah Innis, Bea Cabot, Ryan Cabot, Jonathan Alex Pasternak

**Affiliations:** Department of Animal Sciences, Purdue University, West Lafayette, IN USA

**Keywords:** UOL, UHO, Fetus, IUGR, Swine model

## Abstract

**Background:**

Litter size is a key economic trait in the swine industry, and ongoing selection for this trait has increased ovulation rates without producing a corresponding increase in uterine capacity. Overcrowding, intensifies competition between fetuses, increasing within-litter variation and the occurrence of intrauterine growth restriction (IUGR).

**Methods:**

We utilized a combination of unilateral oviductal ligation (UOL) and unilateral hysterectomy-ovariectomy (UHO), to create apposing biological extremes of intrauterine crowding. A total of twenty gilts including *N* = 7 UHO, *N* = 7 UOL and *N* = 6 unaltered controls (CON) were synchronized and bred via artificial insemination. Embryonic loss and fetal viability were evaluated on gestation day 95. The fetal population intensively phenotyped including morphometric assessment of skull shape.

**Results:**

As expected, UOL significantly reduced litter size, but increased embryonic survival, fetal viability, body weight and uniformity compared to CON. In contrast, UHO significantly increased early embryonic loss, but did not alter fetal viability, body weight or uniformity within litter. UOL increased the absolute weight of all organs compared to UHO and CON, with the exception of the brain (BRN) resulting in a significant reduction in BRN consistent with the established brain-sparing effect. The ratio of skull curve-to-linear length (C: L) was significantly reduced in UOL fetuses and found to be strongly correlated with brain-to-liver weight ratio (BRN: LVR).

**Conclusions:**

The results of this study confirm the negative effects of uterine crowding on fetal growth. They further suggest that contemporary gilts approach the limit of uterine capacity, such that increased crowding results in additional early embryonic loss.

**Supplementary Information:**

The online version contains supplementary material available at 10.1186/s12917-025-04820-x.

## Background

As an important economic trait, litter size remains a primary focus for swine genetic selection in swine. However, selecting for larger litters has increased the number of unthrifty, small or intrauterine growth restricted (IUGR) piglets, which collectively exhibit disproportionately high preweaning mortality [1, 2]. Indeed, contemporary swine are more likely to exhibit severe, naturally occurring IUGR than other domestic animals [3]. One explanation for these effects is the limitation of uterine capacity in dams, referring to the uterus’s restricted ability to support the development of a large number of conceptuses [4–10]. In a crowded uterine environment, conceptuses compete for uterine surface area to attach and undergo placentation. In this context, reduced uterine surface area for placental development may limit nutrient and gas exchange between maternal and fetal systems, resulting in varying degrees of IUGR and substantial within-litter variation in birth weight [11–15]. As such, IUGR has emerged as a crucially important research area in swine. It has supplemental biomedical relevance in the context of human fetal development as pigs area high value biomedical model with the potential to in elucidating both the underlying mechanisms and pre- and post-natal effects [16, 17].

While IUGR occurs along a spectrum, current estimates suggest 20–32% of newborn piglets are affected [18–20], with varying degrees of severity and a stochastic distribution within litter. In order to more directly study IUGR and the effects of uterine crowding in swine, there is a need to develop reproducible methods to generate biological extremes in fetal growth distribution. One approach to inducing IUGR is the unilateral hysterectomy-ovariectomy (UHO) surgery, which involves removing one ovary and the ipsilateral uterine horn, thus increasing uterine crowding and subsequent within-litter variation [21]. This method has been used to study the effect of reduced uterine space on litter size, as a compensatory ovarian response occurs in the remaining ovary, forcing all gametes to develop in the remaining uterine horn [11, 22]. In contrast, a unilateral oviductal ligation (UOL), which involves ligating one oviduct, prevents fertilization of oocytes from the ipsilateral ovary, thereby increasing uterine space per conceptus and lowering the incidence of IUGR [23]. Together, the UHO and UOL may be useful comparative surgical models to induce or reduce IUGR incidence, respectively, in order to better characterize IUGR in this species.

The brain-to-liver weight ratio (BRN: LVR) is widely accepted marker for identifying IUGR, supported by the long-hypothesized brain-sparing effect [9]. When faced with in utero stressors such as reduced oxygen or nutrient supply, brain development is prioritized at the expense of other organs, such as the liver, resulting in non-allometric organ growth [24–26]. However, the utility of this method of IUGR identification is limited due to its terminal nature, so in the previous decade, studies have examined the relationship between IUGR and head shape, aiming to develop non-invasive and rapid methods for identifying IUGR [19, 27–29]. Key morphological characteristics of IUGR piglets include dolphin-like foreheads, bulging eyes, and wrinkles perpendicular to the mouth [28]. While the efficacy of these features in identifying IUGR has been supported by measurement of the BRN: LVR ratio, they require more quantitative measurement for objective assessment. Therefore, the objectives of this study were to (1) validate UOL and UHO surgeries as methods for adjusting uterine crowding in contemporary swine, (2) compare outcomes of different uterine crowding environments at both gilt and fetal levels, and (3) evaluate the utility of the surgical models to reproduce quantitative changes in phenotype.

## Results

### Reproductive characteristics of gilts

Each gilt was used as an experimental unit in gilt-level analysis. A total of 205 fetuses were collected from the twenty gilts in this study, which included nineteen non-viable (non-VIA) fetuses (Table 1). Among the non-VIA fetuses, seventeen were classified as mummified (MUM), with one MUM fetus originating from a UOL litter, ten from control (CON) litters, and six from UHO litters. There was one non-viable (DEAD) fetus from a UHO litter, and one autolyzed (AUT) fetus from a CON litter. No evidence of meconium-staining (MEC), an indicator of in utero stress, was observed in the fetal population from any of the three groups. The proportion of non-VIA fetuses were significantly lower in the UOL group (*P <* 0.05), whereas no significant differences were observed between the CON and UHO groups. The overall ovulation rate remained consistent across all three groups; however, the effective ovulation rate was significantly decreased in UOL gilts (*P <* 0.01). Additionally, the average litter size was significantly reduced in both UOL and UHO groups compared to the CON group (*P <* 0.01). Both embryonic and fetal survival rates were highest in the UOL group and lowest in the UHO group (*P <* 0.01). Two fetuses in the viable (VIA) category were classified as neural tube defects (NTDs), which were excluded from subsequent analyses to prevent misleading conclusions.

**Table 1:** Reproductive characteristics of dams

Parameters	Group			*P*-value
	CON (*n* = 6)	UOL (*n* = 7)	UHO (*n* = 7)	
Total fetus	86	64	55	
VIA	75 (87.21%)	63 (98.44%)	48 (87.27%)	0.037
Non-VIA	11 (12.79%)^a^	1 (1.56%)^b^	7 (12.73%)^a^	
Overall ovulation rate	19.17 ± 3.66	20.29 ± 3.82	20.14 ± 2.41	0.812
Effective ovulation rate	19.17 ± 3.66^a^	9.86 ± 2.79^b^	20.14 ± 2.41^a^	< 0.001
Litter size	14.33 ± 3.01^a^	9.14 ± 2.12^b^	7.86 ± 1.46^b^	< 0.001
Embryonic survival rate (%)	76.03 ± 16.60^b^	93.98 ± 7.56^a^	39.34 ± 7.49^c^	< 0.001
Fetal survival rate (%)	67.79 ± 26.12^b^	91.93 ± 7.59^a^	33.69 ± 9.70^c^	< 0.001

Data are presented as mean ± standard deviation. CON = Control; UOL = Unilateral oviductal ligation; UHO = Unilateral hysterectomy-ovariectomy; VIA = Viable; Non-VIA = Non-viable. ^a, b,c^ Values within a row with different superscripts differ significantly at *P <* 0.05

### Fetal morphology

Each VIA fetus was used as an experimental unit in all fetal-level analyses. In fetal morphology analysis, the average body weight (BW), empty body weight (EBW), crown-rump length (CRL), and girth were significantly greater in UOL fetuses compared to those in the CON group (*P <* 0.05, Table 2), while the BW, EBW, CRL, and girth, were unaltered in the UHO group relative to either CON or UOL. Regarding the coefficient of variation (CV), the UOL litters exhibited the lowest CV in BW, EBW, CRL, and girth (*P <* 0.05). Density and its CV were not significantly different across any of the groups (*p* = 0.27 and 0.92, respectively). There was an increasing trend of skull curve length in the UOL group (*p* = 0.09), whereas skull linear length was significantly increased in UOL group (*P <* 0.05), resulting in a decreased skull curve-to-linear length ratio (C: L) in UOL fetuses compared to CON fetuses (*P <* 0.05). Both UOL and UHO groups exhibited increased snout heights, while skull height was increased only in the UOL group (*P <* 0.01), resulting in significantly increased snout-to-skull height ratio in the UHO group (*P <* 0.01).

**Table 2:** Fetal phenotypes

Parameters	Group			*P*-value
	CON (*n* = 75)	UOL (*n* = 63)	UHO (*n* = 46)	
Body weight (g)	629.11 ± 165.64^b^	861.14 ± 111.39^a^	723.89 ± 196.63^ab^	0.009
Body weight CV	18.07 ± 8.48^a^	8.29 ± 3.64^b^	16.90 ± 6.87^ab^	0.026
Empty body weight (g)	508.04 ± 137.37^b^	700.16 ± 91.79^a^	579.00 ± 162.20^ab^	0.009
Empty body weight CV	18.47 ± 9.07^a^	8.76 ± 3.77^b^	16.91 ± 7.37^ab^	0.044
CRL (mm)	295.48 ± 27.99^b^	326.56 ± 16.97^a^	308.26 ± 28.14^ab^	0.013
CRL CV	7.03 ± 2.99^a^	3.76 ± 1.14^b^	6.61 ± 2.46^ab^	0.036
Girth length(mm)	71.37 ± 7.68^b^	80.35 ± 4.36^a^	73.81 ± 8.15^ab^	0.013
Girth length CV	6.98 ± 3.63^ab^	3.74 ± 0.94^b^	7.39 ± 3.07^a^	0.048
Density (g/cm^3^)	0.92 ± 0.06	0.92 ± 0.06	0.94 ± 0.07	0.271
Density CV	5.26 ± 2.43	5.21 ± 1.75	5.66 ± 2.48	0.922
Head				
Skull curve length (mm)	91.53 ± 4.53	94.70 ± 3.37	93.84 ± 4.19	0.089
Skull linear length (mm)	82.70 ± 5.26^b^	87.74 ± 3.33^a^	85.49 ± 5.20^ab^	0.033
Skull curve-to-linear length	1.11 ± 0.03^a^	1.08 ± 0.01^b^	1.10 ± 0.04^ab^	0.046
Snout height (mm)	42.45 ± 4.29^b^	47.35 ± 2.61^a^	46.31 ± 4.05^a^	0.009
Skull height (mm)	63.18 ± 5.01^b^	68.89 ± 3.10^a^	65.57 ± 4.46^ab^	0.009
Snout-to-skull height	0.67 ± 0.02^b^	0.69 ± 0.02^a^	0.71 ± 0.02^a^	< 0.001

Data are presented as mean ± standard deviation, with coefficient of variation calculated within litter. CON = Control; UOL = Unilateral oviductal ligation; UHO = Unilateral hysterectomy-ovariectomy; CRL = Crown rump length. ^a, b^ Values within a row with different superscripts differ significantly at *P <* 0.05

### Fetal organ weights

**Fig. 1:**
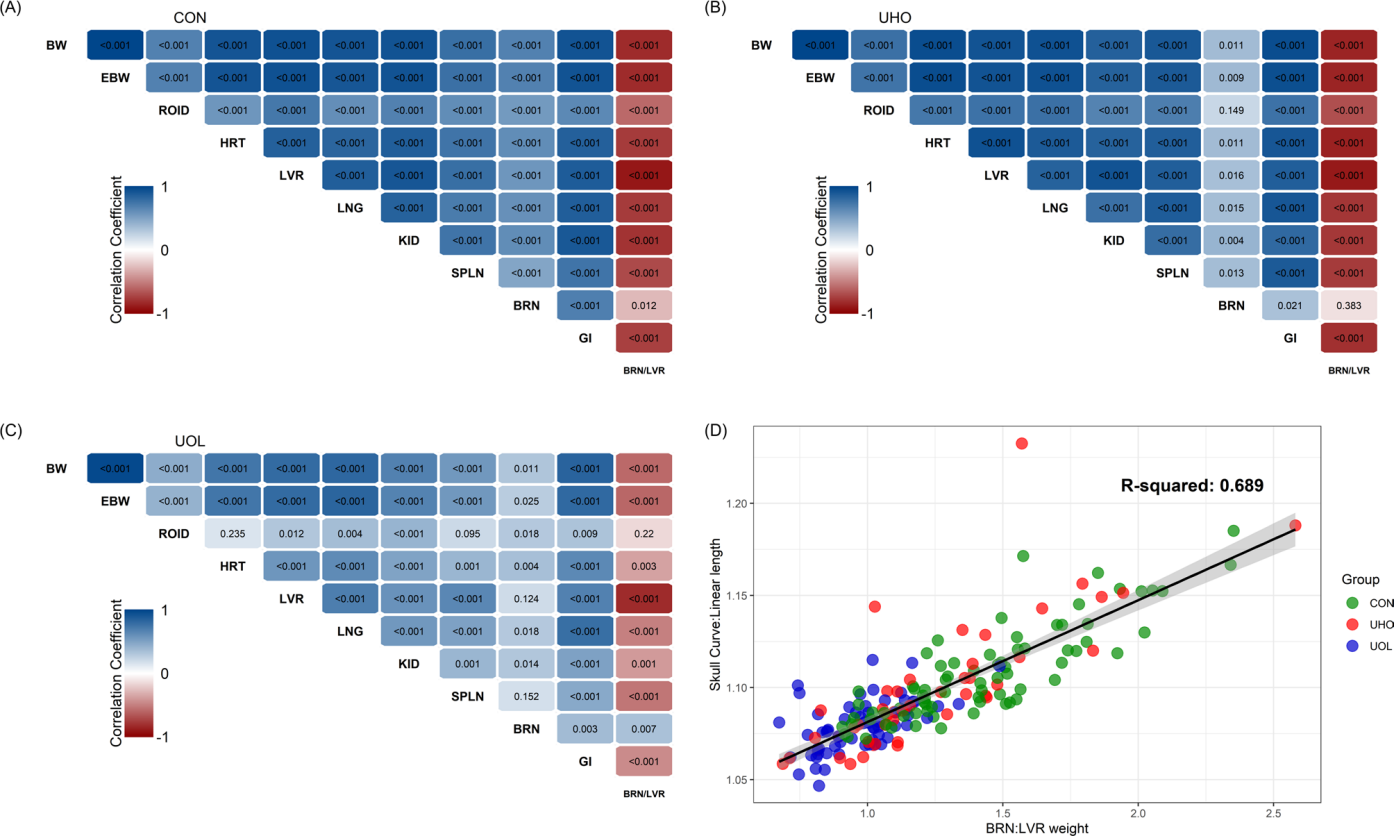
Phenotypic correlations. (**A**) Control. (**B**) Unilateral oviductal ligation. (**C**) Unilateral hysterectomy-ovariectomy. (**D**) Correlation of brain-to-liver weight ratio and skull curve-to-linear length ratio

The weights of key organs were compared across groups (Table 3). Most absolute organ weights were heaviest in the UOL group (*P <* 0.05), except for the brain (BRN), where no significant difference was observed (*P* = 0.191). The BRN: LVR was significantly decreased in UOL fetuses compared to CON fetuses (*P <* 0.01), but unaltered in the UHO group relative to either CON or UOL. Relative organ weights, defined as the ratio of organ weight to BW, were similar between groups, except for BRN and kidneys (KID). The relative BRN weight was diminished in the UOL group compared to the CON group (*P <* 0.05), and the relative KID weight was decreased in both UOL and UHO groups (*P <* 0.01). To assess relationships between organs, correlations among fetal organ weights were analysed within each group (Fig. 1A and C). Overall, the UHO fetuses showed similar correlation patterns to the CON group, while correlations appeared weaker in the UOL group. BW and EBW had a strong positive correlation in all groups, with a correlation coefficient close to 1.00. A positive correlation between BRN and liver (LVR) was observed in CON and UHO groups, but this correlation was less pronounced in the UOL group (*P* = 0.124). The BRN and BRN: LVR were negatively correlated in the CON group, whereas positively correlated in the UOL group. The correlation between BRN: LVR and head shapes was analysed (Fig. 1D). The correlation of determination (R^2^) between BRN: LVR and C: L was 0.689, whereas the R^2^ between BRN: LVR and the snout-to-skull height ratio was 0.206 (data not shown).

**Table 3:** Absolute and relative fetal organ weights

Parameters	Group			
	CON (*n* = 75)	UOL (*n* = 63)	UHO (*n* = 46)	*P*-value
Absolute organ weight (g)				
BRN	20.26 ± 2.39	21.40 ± 1.95	20.98 ± 1.95	0.191
HRT	5.01 ± 1.39^b^	7.00 ± 0.90^a^	5.90 ± 1.74^ab^	0.008
LVR	15.09 ± 4.05^b^	22.40 ± 3.32^a^	18.33 ± 5.08^ab^	0.002
LNG	18.70 ± 5.39^b^	25.58 ± 4.44^a^	20.81 ± 6.77^ab^	0.017
KID	7.13 ± 2.10^ab^	8.74 ± 1.54^a^	6.99 ± 2.24^b^	0.044
SPLN	1.05 ± 0.31^b^	1.40 ± 0.25^a^	1.18 ± 0.39^ab^	0.016
ROID	0.17 ± 0.06^b^	0.22 ± 0.06^a^	0.17 ± 0.05^ab^	0.044
GI	26.34 ± 7.97^b^	35.83 ± 6.23^a^	31.37 ± 9.78^ab^	0.033
BRN: LVR	1.42 ± 0.34^a^	0.97 ± 0.16^b^	1.23 ± 0.37^ab^	0.007
Relative organ weight (g/kg)				
BRN	34.11 ± 7.97^a^	25.20 ± 3.54^b^	31.47 ± 10.26^ab^	0.034
HRT	7.98 ± 0.86	8.17 ± 0.77	8.10 ± 0.63	0.631
LVR	24.09 ± 2.26	26.06 ± 2.49	25.54 ± 3.09	0.243
LNG	29.74 ± 3.22	29.64 ± 2.90	28.39 ± 3.30	0.183
KID	11.29 ± 1.28^a^	10.16 ± 1.35^b^	9.67 ± 1.50^b^	< 0.001
SPLN	1.69 ± 0.43	1.63 ± 0.24	1.63 ± 0.25	0.618
ROID	0.27 ± 0.07	0.25 ± 0.06	0.25 ± 0.06	0.354
GI	41.62 ± 4.52	41.50 ± 3.90	42.90 ± 4.17	0.480

Data are presented as mean ± standard deviation. CON = Control; UOL = Unilateral oviductal ligation; UHO = Unilateral hysterectomy-ovariectomy; BRN = Brain; HRT = Heart; LVR = Liver; LNG = Lungs; KID = Kidneys; SPLN = Spleen; ROID = Thyroids; GI = Gastrointestinal tract. ^a, b^ Values within a row with different superscripts differ significantly at *P* < 0.05

## Discussion

Swine have been subjected to intensive genetic selection to maximize production efficiency, with litter size being a key factor in commercial selection indices. While the resulting increase in litter size may appear beneficial, it has come with some drawbacks, particularly regarding disrupted litter uniformity due to the limitation of uterine capacity [6]. This disrupted within-litter uniformity results in management challenges, particularly for farms that have adopted all-in all-out systems. In such systems, pigs are managed as a single group, and uneven growth rates due to variable birth weights complicates feeding strategies and delays the overall production schedule. Increased within-litter variation also negatively impacts animal welfare, as low birth weight piglets experience higher preweaning morbidity and mortality rates [30]. To better understand the relationship between uterine space and fetal development, we utilized a combination of UOL and UHO surgical procedures to generate biological extremes in crowding. Our results indicate that increasing the uterine space for each conceptus decreases litter variation and classical markers of IUGR. To the best of our knowledge, this is the only study in at least a decade that specifically addresses porcine models of both increased and decreased uterine crowding simultaneously.

Fetuses were distributed across both uterine horns, confirming post fertilization redistribution of the embryos from one oviduct to both uterine horns still occurred regardless of surgical intervention status (See Additional file 1). This is consistent with a previous study utilizing UOL, where 58% of fetuses were found in the uterine horn with the intact oviduct, while 42% were located in the opposite uterine horn [11]. It is essential that embryos are able to migrate between uterine horns, as it allows them to occupy sufficient space for implantation, and this phenomenon happens regardless of uterine length, number of embryos, or number of corpora lutea [31]. In the present study, while embryo distribution across both uterine horns occurred in both UOL and CON gilts, the effective ovulation rate was reduced by 48% in UOL group compared to CON group. Taken together, this suggests that uterine space per fetus was increased in UOL gilts compared to CON.

Fetal mummification, with a prevalence ranging from 1.9 to 6.8% in swine herds, refers to a condition in which dead fetuses become dehydrated and their fetal membranes shrivel, but the fetus remains in the uterus without being aborted [32, 33]. The causes of fetal mummification in swine are varied, including litter size, uterine environment, parity, and the presence of mycotoxins or infectious diseases. In our study, a decrease in the ratio of mummified fetuses was observed in UOL litters (CON 11.6%, UHO 10.9%, UOL 1.6%). This finding agrees with previous studies showing that limited uterine space, increased litter size, and lower average birth weight are more likely to contribute to higher prenatal death and mummification rates [34–36]. Therefore, the decrease in fetal mummification in UOL litters suggests that alleviation of uterine crowding produces more viable fetuses.

Overall ovulation rate was examined to determine whether the surgical procedures caused any unintended disruption to reproductive physiology. In this study, there were no significant differences in overall ovulation rates across the three groups (Table 1). Similar findings have been reported in previous studies, where approximately twice as many ova per uterine horn were observed in UHO gilts compared to CON gilts, resulting in no difference in overall ovulation rate [7, 22]. This can be explained by ovarian compensation, a mechanism where, upon the removal of one ovary, follicle-stimulating hormone (FSH) from the pituitary stimulates the remaining ovary, leading to increased ovarian weight, increased follicle size, and an increased number of large follicles [37–39]. Our data supports the presence of compensatory ovarian response in the UHO group and also suggests that neither surgical procedure disrupted the maternal hypothalamic-pituitary-ovarian axis.

Litter size was decreased in both UOL and UHO groups compared to the CON group (Table 1). Despite a significant reduction in effective ovulation rate in UOL, the absence of a significant difference in litter size between UOL and UHO gilts can be explained by the divergent results in embryonic and fetal survival rates. Both embryonic and fetal survival rates were highest in the UOL litters and lowest in UHO litters. Dziuk (1968) reported that there was little effect of uterine crowding on embryonic survival rates [11]; however, since the average litter size in that study was 9.9, there may have been less uterine crowding compared to the contemporary gilts in the present study. Another previous study suggested that neither the embryonic nor fetal survival rates changed when uterine space per fetus increased, while both rates were decreased when uterine space per fetus was halved [40]. This finding has been supported by other studies conducted in subsequent decades. For example, despite compensated ovulation rates, the uterine size did not increase proportionally, leading to reduced fetal survival and smaller litter sizes at gestation day 86 in UHO gilts [22]. Similarly, Pere et al.(1997) observed decreased embryonic and fetal survival rates in UHO gilts and concluded that reduced uterine capacity influenced the mortality rate of conceptuses, particularly during the embryonic stage [7]. The aforementioned results are consistent with the findings of the current study, where both embryonic and fetal survival rates were decreased in response to uterine crowding in the UHO group. It is worth noting the average litter size observed in the CON gilts was below what is often observed in the most prolific maternal lines. While relevant to the terminal crosses that make up the majority of the production system, the present results may underestimate the impact of uterine crowding observed in purebred Landrace or Large White populations.

Of note, two NTDs fetuses were observed in the same UHO litter. NTDs comprise a broad range of developmental malformations during neurulation that affect the central nervous system, including the brain, spinal cord, and surrounding tissues [41]. In pigs, neurulation occurs from gestation day 14 to 18 [42, 43]. On the other hand, crowding does not become as issue until after gestation day 25 [11]. Thus, the NTDs fetuses were classified as VIA fetuses, as it is unlikely that uterine crowding was the cause. However, their data were excluded from fetal-level morphological analysis, as their malformed brains and head shapes could have led to misleading conclusions.

The decreased CV of within-litter variation across multiple metrics in the UOL group suggests that litters were more uniform in the UOL group (Table 2). In contrast, the lack of significant differences of morphological features between CON and UHO groups suggests that uterine crowding is maximized in contemporary gilts. To determine if body muscle and fat composition differed between groups, we compared fetal density, and no significant differences were observed across the groups. Previous studies have suggested that smaller or IUGR pigs have different muscle-to-fat ratio. For instance, fetuses from modestly crowded litters were found to have fewer secondary muscle fibres, impeding fetal development and resulting in lower BW [17, 23]. One possible explanation for the difference between our study and previous studies is that total fat in newborn pigs, accounting for only around 2% of their body weight [44], may not be significantly affected by uterine crowding. Additionally, unlike previous studies that compared IUGR versus non-IUGR fetuses, we compared crowded versus less crowded litters, suggesting that average density might have been compensated for by non-IUGR fetuses within each group or litter due to the within-litter variation. Further investigations are needed to better understand fetal body composition under uterine crowding conditions.

In normal fetuses, BW, EBW, as well as the weight of the LVR, heart (HRT), lungs (LNG), KID, and gastrointestinal tract (GI) are expected to increase cubically throughout gestation, while BRN weight increases linearly between gestational days 45 to 110 [45]. We collected fetal organ samples at gestation day 94–96, and the differences in organ weights between groups support our hypothesis that uterine crowding affects fetal organ development. Previous studies have consistently identified brain weight as stable regardless of developmental trajectory [12, 16, 23, 29, 46]. However, there is some disagreement in the literature as to which organs may experience asymmetrical growth. Widdowson (1971) found that in newborn pigs, the muscle, LVR and KID of runts were the most growth restricted, while the HRT, spleen (SPLN), and stomach were less impacted [12]. Similarly, Bauer et al. (1998) investigated the variation in body and organ weights in 1-day-old pigs, reporting that KID, endocrine glands (adrenal glands, thymus, thyroid gland; ROID), and other abdominal organs (LNG, LVR, SPLN, pancreas) exhibited the greatest weight variation, while the HRT varied to a lesser extent [16]. In contrast, Lynegaard et al. (2019) reported that the LVR, LNG, and adrenal glands were less impacted in IUGR newborns, like the BRN [29]. Given that these studies were conducted with newborn pigs, the differing results in our study may stem from the gap between gestation day 95 and birth. As the growth rate of the fetus and most organs continues to increase with gestational age, it is possible that the impact of altered uterine space may be exacerbated closer to term. However, by gestational day 91–96, fetal BRN weight is nearly the same as at full-term, while other organs, such as the LVR, KID, HRT, and SPLN increase in weight by approximately 2.5 times during the last days before farrowing [12]. Additionally, we analysed the correlation of BW, EBW, and individual organ weights (Fig. 1). The correlation coefficient between BW and EBW was nearly 1.00 in all groups, suggesting that total internal organ mass was not changed regardless of groups. Since absolute BRN weight was consistent across groups (Table 3), other organs may have developed disproportionately under different crowding conditions. The similarity in overall correlation patterns between CON and UHO fetuses, as compared to the UOL group, suggests that (1) uterine crowding has likely reached its maximum in contemporary gilts, and (2) relieving uterine crowding through UOL surgery alters fetal organ developmental rates.

The BRN: LVR is currently the most reliable method for identifying IUGR pigs, as it indicates whether asymmetric growth occurred during fetal development. However, the invasive and terminal nature of employing this identification method limits its use in longitudinal studies, thus highlighting the need for non-invasive methods to identify IUGR pigs in vivo. Some studies have suggested that IUGR pigs exhibit distinct head morphology, such as dome-shaped heads [13] or dolphin-like foreheads [28]. However, the limitation of these studies is that they relied on subjective methods, such as author-defined scoring systems, rather than quantifiable measurements. Thus, we measured various head shape parameters of fetuses in lateral recumbency to assess their potential use as a non-invasive identification method of IUGR. The selected measurements were highly repeatable, with the CV between each side ranging from 0.58 to 0.77 (data not shown). Of the measurements evaluated, a strong positive correlation was observed between the BRN: LVR and the C: L (Fig. 1D, R^2^ = 0.689). Given the strong correlation, this head shape parameter may be useful for non-invasively identifying IUGR animals. Additionally, in the same figure, the UOL data shows a greater extent of clustering relative to other groups, suggesting more uniform litters in this group. Considering these results, further investigation into head shape should be performed to validate its definitive use as a quantitative and non-invasive method for identifying IUGR pigs.

## Conclusion

Our data suggests that artificially decreasing uterine crowding increases embryonic survival, fetal survival, and overall fetal growth, whilst decreasing the incidence of IUGR and the severity of within litter variation. In contrast, we showed that further increases in uterine crowding exacerbate embryonic loss. Additionally, artificially increasing uterine crowding does not alter fetal growth by gestational day 95 relative to controls, suggesting uterine crowding may be maximized in contemporary terminal cross gilts. These findings highlight not only the trade-off between increased litter size and uniform, viable litters, but the significant impact of uterine crowding on fetal growth. Future research is required to fully elucidate the causation, mechanisms and outcomes of IUGR.

## Materials and methods

### Animals and surgical procedures

**Fig. 2:**
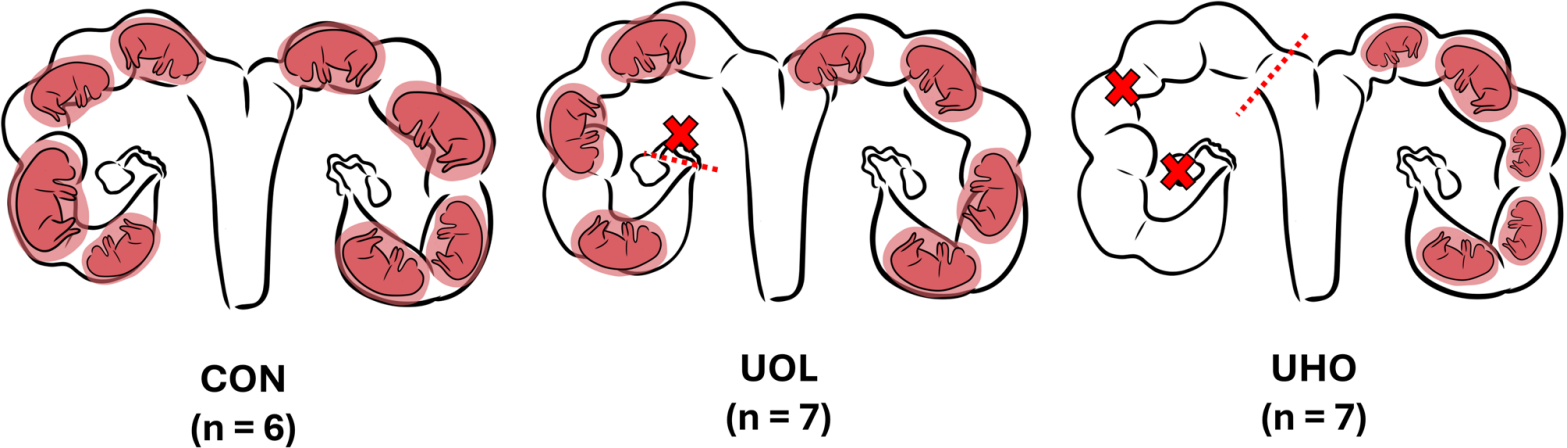
Gilts group by surgical method

The sample size was determined based on previous studies that utilized UOL and/or UHO surgical procedures [47–49]. A total of twenty Landrace x Large White gilts (6–8 month olds) were selected from the Animal Sciences Research and Education Center breeding herd at Purdue University and were randomly allocated to control (CON; *n* = 6), UOL (*n* = 7), or UHO (*n* = 7) in groups of equivalent age. All gilts were confirmed as cyclic and either UOL or UHO surgery was performed in each corresponding group (Fig. 2). Anaesthesia was induced with intravenous injection of 5 mg/kg ketamine and 1 mg/kg xylazine and maintained with inhalation of 2–5% isoflurane in oxygen with a flow rate of approximately 2 L per minute. A 4 cm midline abdominal incision was made to expose the surgical site. For the UOL operation, a single ovary and its ipsilateral oviduct were exposed up to the utero-tubular (UT) junction. A 2–3 cm portion of the oviduct was clamped using an angiotribe, and the clamped section of oviduct tied off with absorbable chromic gut suture and removed. In the UHO operation, a single uterine horn and the attached ovary were exteriorized. The broad ligament under the ovary was clamped, and all vessels from the ovary to the uterine bifurcation were tied off and clamped with an angiotribe. The uterine horn was clamped approximately 1–2 cm above the uterine bifurcation, followed by tissue removal commencing from either the uterine horn or ovary until the entire ovary and uterine horn on one side were removed. After confirming hemostasis in both procedures, the remaining reproductive tissue was rinsed with 37℃ sterile phosphate-buffered saline (PBS) before being returned to the abdominal cavity. The incision was closed with three layers of absorbable suture. Throughout all procedures, the reproductive tract was kept moist with 37℃ sterile PBS to minimize adhesions.

All gilts were moved to the same room, randomly allocated to individual pen regardless of surgery, monitored twice daily for 3–4 days post-surgery, and given at least a 21-day recovery period before estrus synchronization and breeding. After recovery, estrus was synchronized in all animals using oral Matrix (Altrenogest, 15 mg/day) for 14 days. Following progestin withdrawal, gilts were heat checked twice daily to identify a lordosis response to fence line contact with a boar. Gilts were bred by intracervical artificial insemination (AI) 12 h after the first estrus, and every 24 h after until estrus behaviour was no longer observed. Pooled commercial semen from Duroc boars was used for AI. Around 30 days post-AI, pregnancy was confirmed by transcutaneous ultrasound. All gilts had ad libitum access to water and were fed standard gestation diets composed of 51.4% corn, 13.7% soybean and 30% dried distillers grains and formulated to meet nutritional requirements based on the NRC guidelines [50] including 3272 Kcal/kg metabolizable energy with 18.6% crude protein. All animal work, including surgery, was conducted in strict accordance with Institutional Animal Care and Use Committee (IACUC) guidelines and were approved by the Purdue Animal Care and Use Committee (Protocol # 1222002327).

### Sample collection and fetal morphometry

At 94–96 gestation days, gilts were electrically stunned and euthanized via exsanguination. The gravid uterus was then recovered and linearized for dissection, with fetuses and their corresponding placentas sequentially removed from each uterine horn to preserve their location relative to the utero-tubular junction. Viable fetuses were categorized as previously defined in [51], with those exhibiting pulsations within the umbilical cord identified as VIA, and further assessed for evidence of meconium staining or developmental defects. The remaining fetal population including dead, autolyzed, and mummified fetuses were classified as non-viable.

All VIA fetuses were rapidly exsanguinated via the axillary artery. Both the left and right profiles of each fetus were imaged and recorded with individual ID tags to allow for later phenotypic analysis. Fetal volume was determined by water displacement, BW was measured using a scale with precision to four decimal places in kilograms, and the two measures combined to determine body density. Fetal organs, including BRN, HRT, LVR, LNG, KID, SPLN, ROID, and GI, were collected and weighed using a scale with precision to three decimal places in grams. Finally, EBW were measured, which included the head and legs but excluded the organs that were previously collected.

**Fig. 3:**
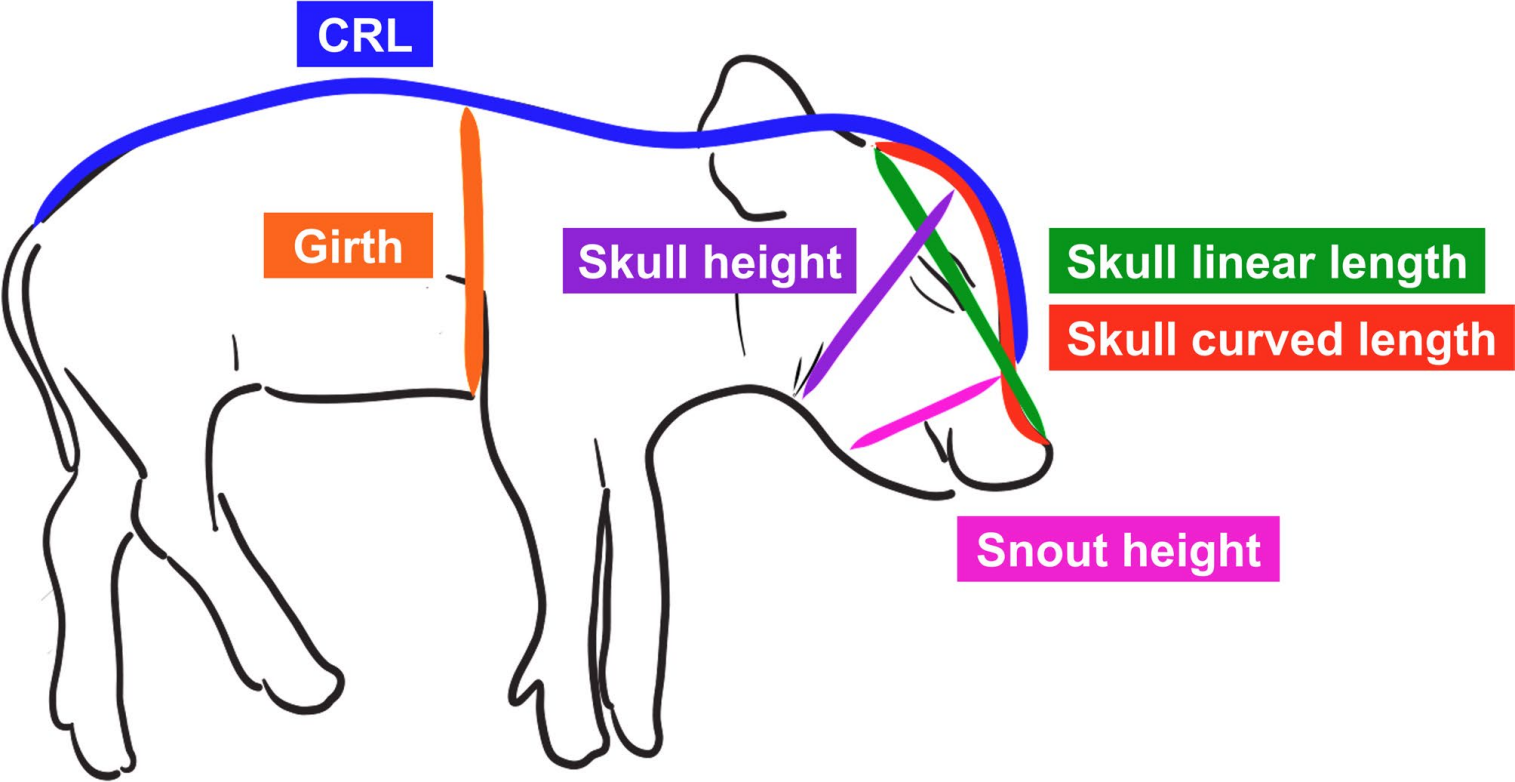
Fetal morphology measurement. CRL = Crown rump length

Using a custom ImageJ software, CRL, girth, and head shape measurements were taken from both the left and right profiles of each fetus, with a standardized ID tag serving as a calibrator reference (Fig. 3). CRL was defined as the distance from the base of the snout to the tail head along the dorsal side of the body, while girth was measured from the chest to the spine just behind the front legs. Skull measurements included skull length, snout heigh, and skull height. The skull linear length was the straight-line distance from just behind the tip of the snout to the ear, and the skull curve length followed the curvature of the skull between these two points. Snout height was measured as the distance from the base of the snout to the jaw, and skull height was measured from the crown to the front of the jowl, touching the caudal edge of the eye. Final fetal morphology data were calculated by averaging both side measurements and the CV of each parameter was calculated within litter.

### Ovulation rates and conceptus survival rates

The number of ovarian corpora lutea (CL) on the maternal ovaries were manually counted during necropsy to determine the ovulation rate of each gilt. The overall ovulation rate represents the total number of ovulated oocytes, while the effective ovulation rate refers to the number of ovulated oocytes available for fertilization [23]. Early embryonic mortality describes the loss of embryos prior to the fetal stage, typically around gestation day 30 [8]. Due to constraints on animal availability, we were unable to collect data at this time point. Instead, we estimated embryonic and fetal survival rates using the following equations:

$$\begin{array}{c}\:Fetal\,survival\,rate\:\left( \% \right) = \\\:\left( {\frac{{VIA\,fetus}}{{Effective\,ovulation\:rate\:}}} \right) \times \:100\end{array}$$

$$\begin{array}{c}\:Early\,embryonic\,survival\,rate\,\left( \% \right) = \:\\\left( {\frac{{Total\,fetus}}{{Effective\,ovulation\:rate\:}}} \right) \times \:100\end{array}$$

### Data analysis

All data were statistically analysed using R software (R version 4.3.1). A linear model and linear mixed model with random effect for litter were employed to analysed gilt and fetal data using the nlme package (Pinheiro and Bates, 2000), followed by a least-square means post hoc test using the emmeans package (Lenth, 2023). Categorical data, such as the number of VIA and non-VIA fetuses, were analysed using the Chi-squared test. Spearman’s rank correlation was applied to assess the correlation of fetal organ growth. Data visualization was conducted using the ggplot2 package (Wickham, 2016). The significance threshold for all analyses was set at *P <* 0.05, with 0.05 < *P <* 0.10 defined as a trend.

## Electronic supplementary material

Below is the link to the electronic supplementary material.

Supplementary Material 1

## Data Availability

The raw data associated with this study are available only upon reasonable request to the corresponding author.

## Abbreviations

UOL: Unilateral oviductal ligation
UHO: Unilateral hysterectomy-ovariectomy
VIA: Viable
Non-VIA: Non-viable
DEAD: Stillborn
MUM: Mummified
AUT: Autolyzed
NTDs: Neural Tube Defects
IUGR: Intrauterine growth restriction
BW: Body weight
EBW: Empty body weight
BRN: LVR: Brain-to-liver weight ratio
BRN: Fetal brain
HRT: Fetal heart
LVR: Fetal liver
LNG: Fetal lungs
KID: Fetal kidneys
SPLN: Fetal spleen
ROID: Fetal thyroid glands
GI: Fetal gastrointestinal tract
C: L: Skull curve-to-skull linear length ratio
