# Supplementary material for: Surgical alteration of uterine space influences embryonic loss and fetal growth in the contemporary pig
Source: BMC Vet Res. 2025 May 20;21:360. doi: 10.1186/s12917-025-04820-x (PMC12090578; doi:10.1186/s12917-025-04820-x)
Supplement: Supplementary file 1 — Supplementary Material 1 [file 12917_2025_4820_MOESM1_ESM.pdf]

Gilt	Group	Left_CL	L1	L2	L3	L4	L5	L6	L7	L8	L9	L10	L11	R11	R10	R9	R8	R7	R6	R5	R4	R3	R2	R1	Right_CL
			**Fetal position**																						
Z21	CON	9	M	V	V														M	V	V	V	V	V	10
D19	CON	9	V	V	V	V	V	V	V					V	V	V	V	V	V	V	V	V	V	V	14
D6	CON	15	V	V	V	V	M								A	V	V	M	M	M	M	M	M	V	9
Y12	CON	8	V	V	V	V	V	V											V	V	V	V	V	V	8
W4	CON	9	V	V	V	M	V	V	V	V						V	V	V	V	V	V	V	V	V	9
W10	CON	8	V	V	V	V	V	V	V									V	V	V	V	V	V	V	7
K16	UOL	7	V	V	V																V	V	V	V	9†
L11	UOL	14	V	V	V	V	V	V											V	V	V	V	V	V	11†
K13	UOL	7	V	V	V	M	V																V	V	10†
K9	UOL	10	V	V	V	V	V	V													V	V	V	V	10†
K18	UOL	13	V	V	V	V												V	V	V	V	V	V	V	13†
K7	UOL	10	V	V	V	V													V	V	V	V	V	V	9†
K29	UOL	8	V	V	V																V	V	V	V	11†
U6	UHO	20	V	V	V	V	M	M																	-
U11	UHO	22	V	V	M	V	V	V	V																-
V3	UHO	21	V	V	V	V	V	V	V	V	V	V													-
L8	UHO	22	V	V	V	V	V	V	V	V	V														-
L2	UHO	21	V	V	V	N	V	N	V																-
W1	UHO	15	V	V	V	S	M	M	V																-
C12	UHO	20	V	V	V	V	V	M	V	V	V														-

† oviductal ligation side

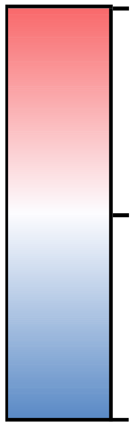

Additional file 2. Spatial distribution and preservation status in uterine horn. Color scale indicates BRN:LVR ratio.
V = Viable; S = Stillborn; M = Mummification; A = Autolyzed; N = Neural tube defects.
